# Supplementary material for: Improving Widescale Monitoring of Ectoparasite Presence in Northern Canadian Wildlife with the Aid of Citizen Science
Source: Insects. 2022 Apr 12;13(4):380. doi: 10.3390/insects13040380 (PMC9025924; doi:10.3390/insects13040380)
Supplement: Supplementary file 1 [file insects-13-00380-s001.zip › insects-1652871-supplementary/Chenery_et_al_SuppMat/SuppMatS1_YWTMP_sample_kit.pdf]

Chenery et al., 2022. Improving widescale monitoring of ectoparasite presence in northern Canadian wildlife with the aid of Citizen Science.

Supplementary materials S1

Yukon Winter Tick Monitoring Project Sample Kit

Description and template materials for the Yukon Winter Tick Monitoring Project (YWTMP) hide sample kit.

Collecting a sample from the areas of known high tick-abundance on an animal, namely the neck and shoulder region or rump, is a common means of assessing winter tick presence (Samuel, 2004; Sine et al., 2009). This kit is designed to obtain a 40 x 20cm section of moose or caribou hide from the right shoulder of the animal, so that it can be visually assessed for winter ticks (*Dermacentor albipictus*).

Note that full host burden (full body tick density estimate) cannot be assessed from this method, which provides a coarse approximation of relative tick numbers per host and presence only data.

Materials needed

Large Ziploc™ bag (26.8 x 27.3cm)
Submission details label (*Appendix A)*
Nitrile gloves (x1 pair, Large size)
Paper template: sample location template & instructions (*Appendix B)*

Kit assembly

1. Label Ziploc™ bag with submission label. Ensure that unique identifier is written into the Sample Kit ID field, and recorded in the lab spreadsheet.
2. Print double-sided paper instructions and template and trim excess paper following guidelines.
3. Place template and one pair of nitrile gloves inside the bag and seal.

Literature cited:

Samuel, W. M. (2004). White as a Ghost: Winter ticks and moose. Natural History Series. Alberta: Federation of Alberta Naturalists.
Sine, M., K. Morris and D. Knupp. (2009). Assessment of a line transect field method to determine winter tick abundance on moose. Alces 45: 143–46.

Section A. Submission Kit Label

Sample kit ID: ____________________ Yukon

Kill date: ______________________________________

Species *(circle)*: CARIBOU MOOSE

Sex *(circle)*: MALE FEMALE

Kill location *(Latitude/Longitude - please be as specific as possible)*: __________________________________

____

Section B. Hide sample kit template and instructions (side 1)

Instructions:

Yukon Winter Tick Monitoring Project

Turn over for Template

[OFFICE USE]

Within 6 hours of kill

Step 1. CUT SAMPLE

Cut 1 piece of hide approx. 40x20cm (use template)

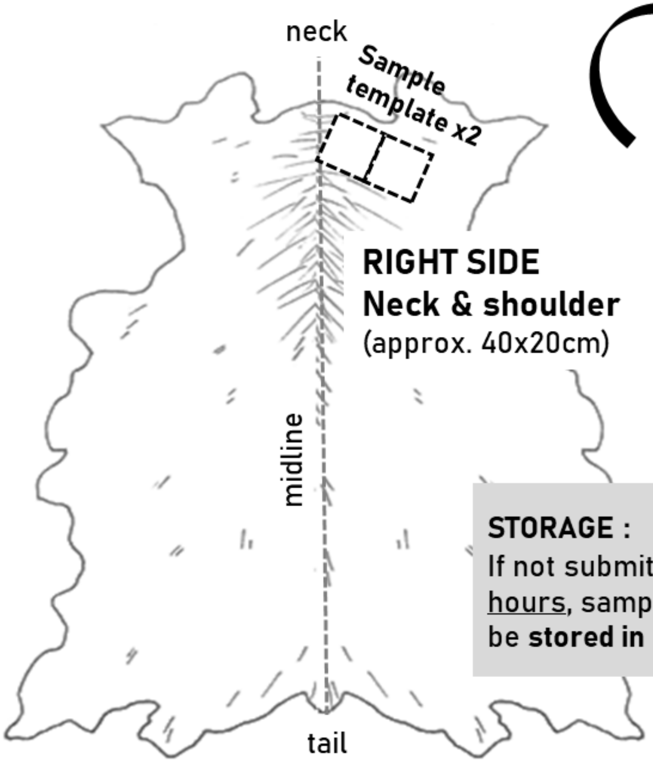

RIGHT SIDE
Neck & shoulder
(approx. 40x20cm)

Step 2. FOLD SAMPLE

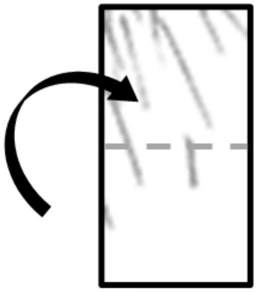

STORAGE :
If not submitting <u>within 24 hours</u>, sample bags can be **stored in the freezer.**

Step 3. BAG SAMPLE

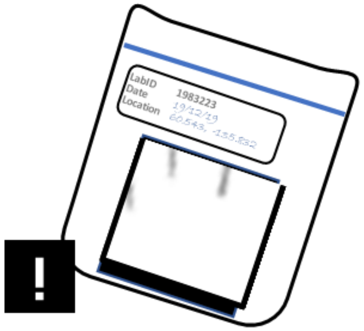

Please ensure bag is <u>fully sealed</u>, and that you have completed the label on the sample bag, including:

- Kill DATE
- Kill LOCATION*

** Please be **as specific as possible**, as this helps us to find the ticks in the landscape.*

- Note - all information received will be kept confidential.

Printing instructions :

- print double-sided
- cut this edge off
- fold so that "Instructions" title is clearly visible
- place leaflet in sample bag.

Section C. Hide sample kit template and instructions (side 2)

Yukon
Winter Tick
Monitoring Project

Turn over for full instructions

Sample Template

Approx. 20cm

Cut approx. 2x size (40 x 20cm)

RIGHT SIDE

neck & shoulder region

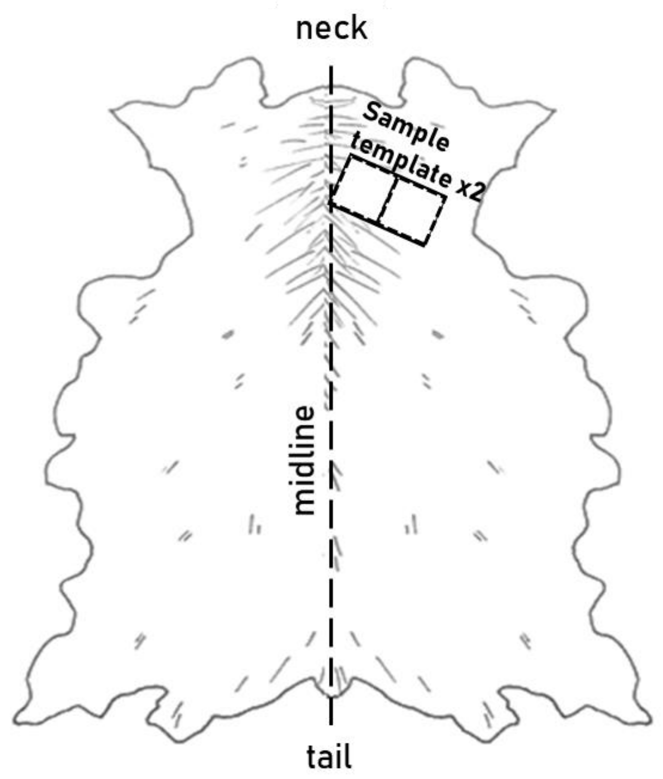

Approx. 20cm
