# Supplementary material for: Improving Widescale Monitoring of Ectoparasite Presence in Northern Canadian Wildlife with the Aid of Citizen Science
Source: Insects. 2022 Apr 12;13(4):380. doi: 10.3390/insects13040380 (PMC9025924; doi:10.3390/insects13040380)
Supplement: Supplementary file 1 [file insects-13-00380-s001.zip › insects-1652871-supplementary/Chenery_et_al_SuppMat/SuppMatS2_YWTMP_sampling_protocol.pdf]

Chenery et al., 2022. Improving widescale monitoring of ectoparasite presence in northern Canadian wildlife with the aid of Citizen Science.

Supplementary materials S2

Yukon Winter Tick Monitoring Project Sampling Protocol

Section A: Sample processing datasheet
Section B: Sample transect template

Sampling methodology to assess winter tick presence and count from samples submitted as part of the Yukon Winter Tick Monitoring Project Hide Submissions Scheme 2019 and 2020.

This method adapts the sub-sampling method used by Sine et al. (2009) to assess hide samples of moose for winter ticks by building on the existing process used by Environment Yukon's Animal Health Unit (Kuba et al., 2016). An intensive visual hide transect method is used on a smaller hide sample (approximately 20cm x 40cm) taken from the right midline and shoulder of the hide. It allows greater manoeuvrability of the hide sample piece to include assessment under a dissecting microscope or using a magnifying sheet.

Equipment / materials needed

- Defrosted hide sample to be processed (note: defrosting takes approximately 8-12 hours)
- Hide sample processing datasheet & pen for notetaking *(Section A)*
- Measuring tape or metre ruler
- Printed hide sample transect template (optional, *Section B*)
- Plain white paper (A3)
- Knitting needle
- 10x magnifier or microscope
- Headlamp or other bright overhead light
- Personal Protective Equipment (lab coat, nitrile gloves)

Methods

1. Collect all materials and equipment needed for processing. Hide samples should be removed from the freezer and thoroughly defrosted, 24 hours in advance. It is important to leave the hide sample in its original bag during defrost so that the bag and hide piece can be processed together.
2. Remove sample from Ziplock™ bag, taking care to note the collection information from the bag label on your datasheet. Set aside for step 4.
3. Lay the sample on a flat surface in a well-lit area, hair side up, with the midline on the left (if visible – see Figure S1). Note the midline should be identifiable by longer hairs along one edge or corner of the sample). Use the transect templates under the top and bottom edges of the sample. This will help to keep transect sampling effort consistent.

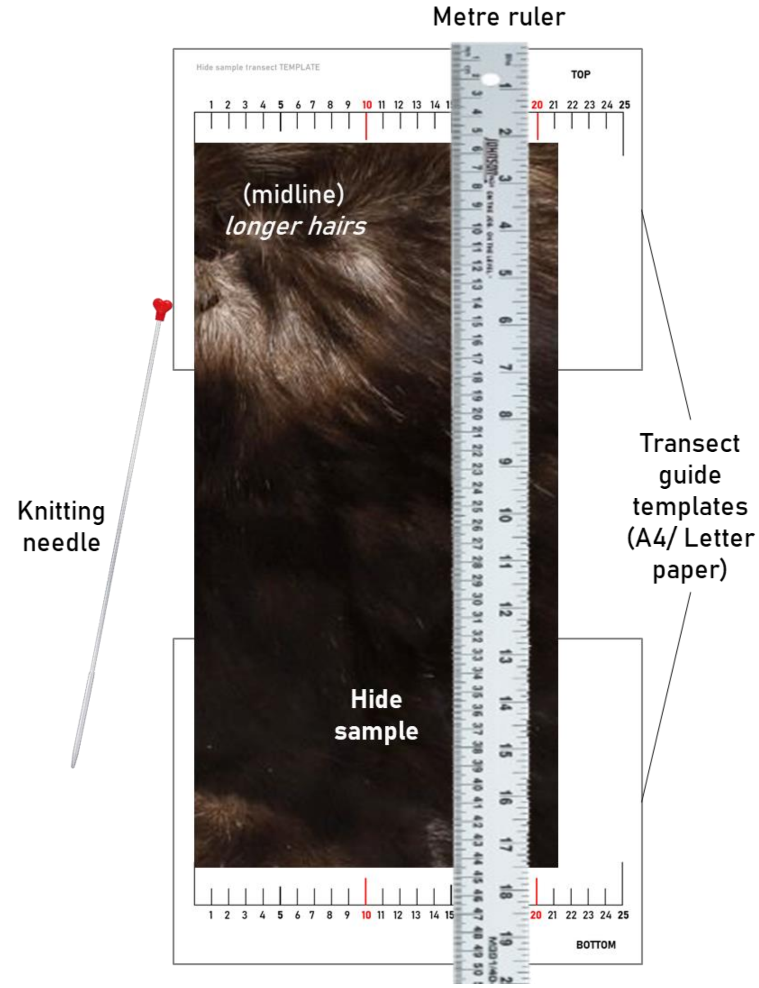

Figure S1.
Suggested set up for processing hide sample pieces.
Using the transect guide templates helps to ensure transects are straight, so that the sampling is of equal effort throughout. The same gauge (width) knitting needle should be used for all samples each season.

4. Next, lay the Ziplock™ bag over a piece of plain white paper to improve visibility. Carefully check for any loose ticks – especially under the Ziplock™ fastening, bag corners or any other creases. Note the number of loose ticks on the datasheet, if applicable.
5. Using the measuring tape or ruler, measure the width and length of the hide sample piece and note on the datasheet.
6. Now complete a visual hide transect check, starting 1cm in from the left-hand edge of the sample. Use a headtorch if necessary, to ensure the sample is fully visible. Each transect should be for the full length of the sample, taking care to part the hair with the knitting needle to expose the skin beneath (see Kuba et al., 2016 for a full description of method). A magnifying sheet or microscope can also be used to ensure larvae are not missed. Cross off transect numbers on the datasheet as they are completed, to help keep a track of each line. Add extra numbers as necessary.
7. If ticks are found, note the transect number, life stage and engorgement status of the tick (Figure S2) on the relevant line of the datasheet.
8. Continue sampling each transect line at 1cm intervals until the whole hide piece has been completed.

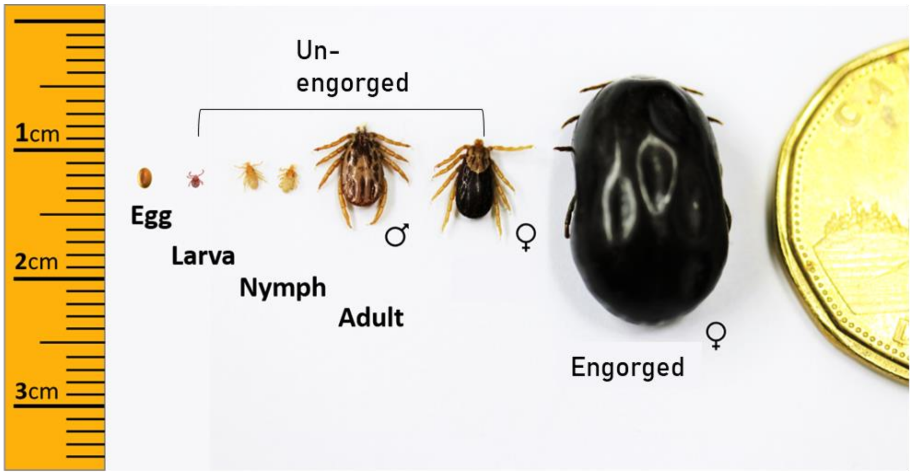

Figure S2.
Winter tick life stages and engorgement statuses.
Larvae are the only life stage to have 6 legs (3 pairs). All other life stages have 8 legs (4 pairs). Adult male winter ticks will never be fully engorged. Fully engorged adult females may look pale grey/brown in colour.
(Images: E. Chenery, 2019)

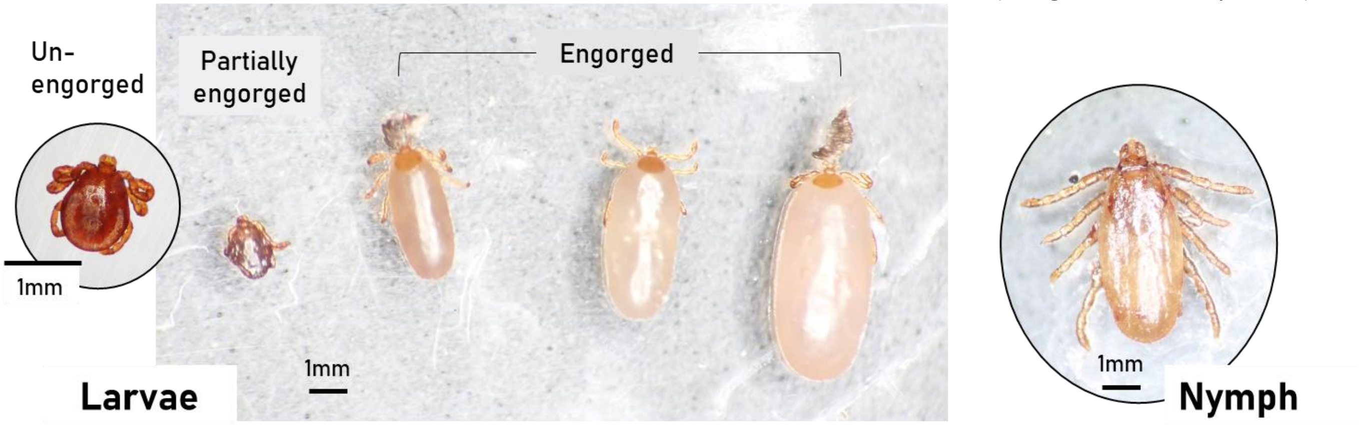

9. Complete the datasheet with the total number of transects used and total the number of ticks across transects, including the 5 transect table on the back of the sheet. If no ticks were detected, make sure to write "0" in the total ticks column (do not leave blank).
10. Hide pieces and bags can be disposed of. Keep tick specimens in ethanol if uncertain of their identification. Be sure to label any specimens clearly with the HIDE and Lab-ID number.

The datasheet should be entered electronically, updating tick totals on the Winter_Tick_Hide_Samples_2011-present.xls using the 5 transect totals, and the full dataset on the Hide_Samples tab of the workbook.

Literature cited:

Kuba, K., M. Larivee, E. Kloppers, R. Ward, R. Florkiewicz, M. VanderKop, & N. J. Harms. (2016). Hair transect sampling method trials for monitoring winter tick (*Dermacentor albipictus*) burdens on elk (*Cervus elaphus*) hides in Yukon. Yukon Fish and Wildlife Branch Report TR-16. Whitehorse, Yukon, Canada.

Sine, M., K. Morris, & D. Knupp. (2009). Assessment of a line transect field method to determine winter tick abundance on moose. *Alces*, *45*(October), 143–146.

Section A.
Hide Sample processing datasheet

WINTER TICK - HIDE SAMPLE KIT PROCESSING DATASHEET

Date processed DD/MM/YY

Processed by initial. Surname

HIDE-ID	

(circle)

Species	moose	caribou
Sex	M	F

Kill date DD/MM/YY

Kill Location

Lat (N)

Lon (W)

HIDE PIECE *(e.g. damage/ hair missing, other)*	L (cm)	W (cm)

	Unengorged				*Engorged*		
Transect no.	L	N	AM	AF	L	N	AF
1							
2							
3							
4							
5							
6							
7							
8							
9							
10							
11							
12							
13							
14							
15							
16							
17							
18							
19							
20							
TOTAL							
	L	N	AM	AF	L	N	AF
	Unengorged				*Engorged*		

Loose ticks (no.)	L	N	AM	AF
uneng.				
engorged				

Transects total

(Space for additional transects overleaf)

WINTER TICK - HIDE SAMPLE KIT PROCESSING DATASHEET

(Continued from p1. additional transects if needed)

	Unengorged					*Engorged*		
	L	N	AM	AF		L	N	AF
Transect no.								
TOTAL								
	L	N	AM	AF		L	N	AF
	Unengorged					*Engorged*		

For Winter_Tick_Hide_Submissions.xls

Number of transects from p1 [ ]

Divide by 5

Round down <.5

Round up >=.5

Using totals from the full transect list overleaf, sum ticks to create 5, even transects.

e.g. For a total of 20 transects, (20/5=4) sum ticks in transects in groups of 4:
1-4, 5-8, 9-12, 13-16 and 14-20.

	Sum transect no.		*Unengorged*				*Engorged*		
	from	to	L	N	AM	AF	L	N	AF
R1									
R2									
R3									
R4									
R5									

Section B.
Hide sample transect template

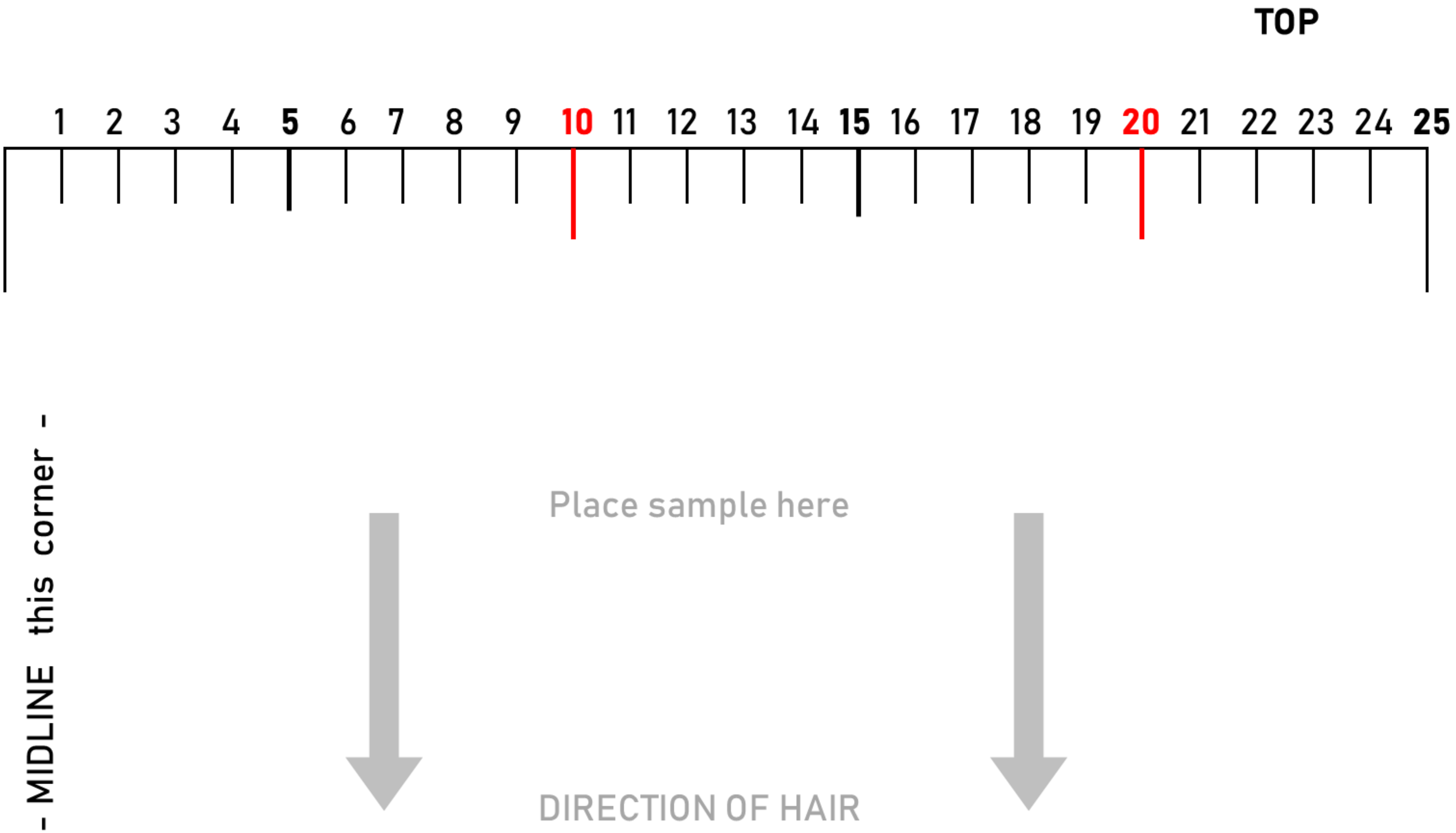

- MIDLINE this edge -

Place sample here

DIRECTION OF HAIR

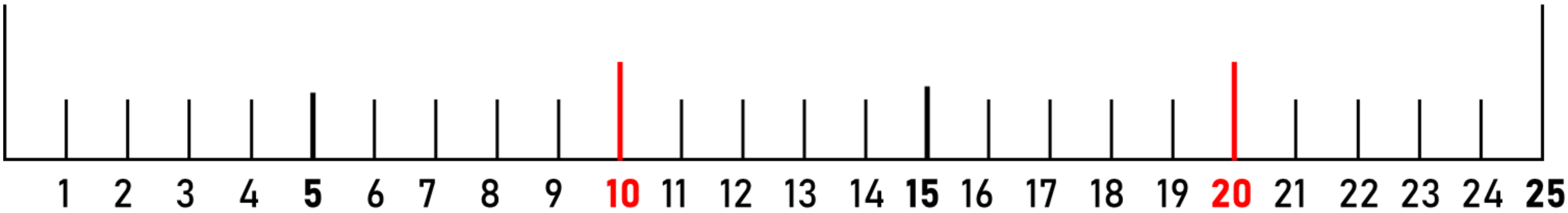

BOTTOM

END
