# Supplementary material for: Improving Widescale Monitoring of Ectoparasite Presence in Northern Canadian Wildlife with the Aid of Citizen Science
Source: Insects. 2022 Apr 12;13(4):380. doi: 10.3390/insects13040380 (PMC9025924; doi:10.3390/insects13040380)
Supplement: Supplementary file 1 [file insects-13-00380-s001.zip › insects-1652871-supplementary/Chenery_et_al_SuppMat/SuppMatS3_Hide_submissions_ALL_by_GMS.pdf]

Chenery et al., 2022. Improving widescale monitoring of ectoparasite presence in northern Canadian wildlife with the aid of Citizen Science.

Supplementary materials S3

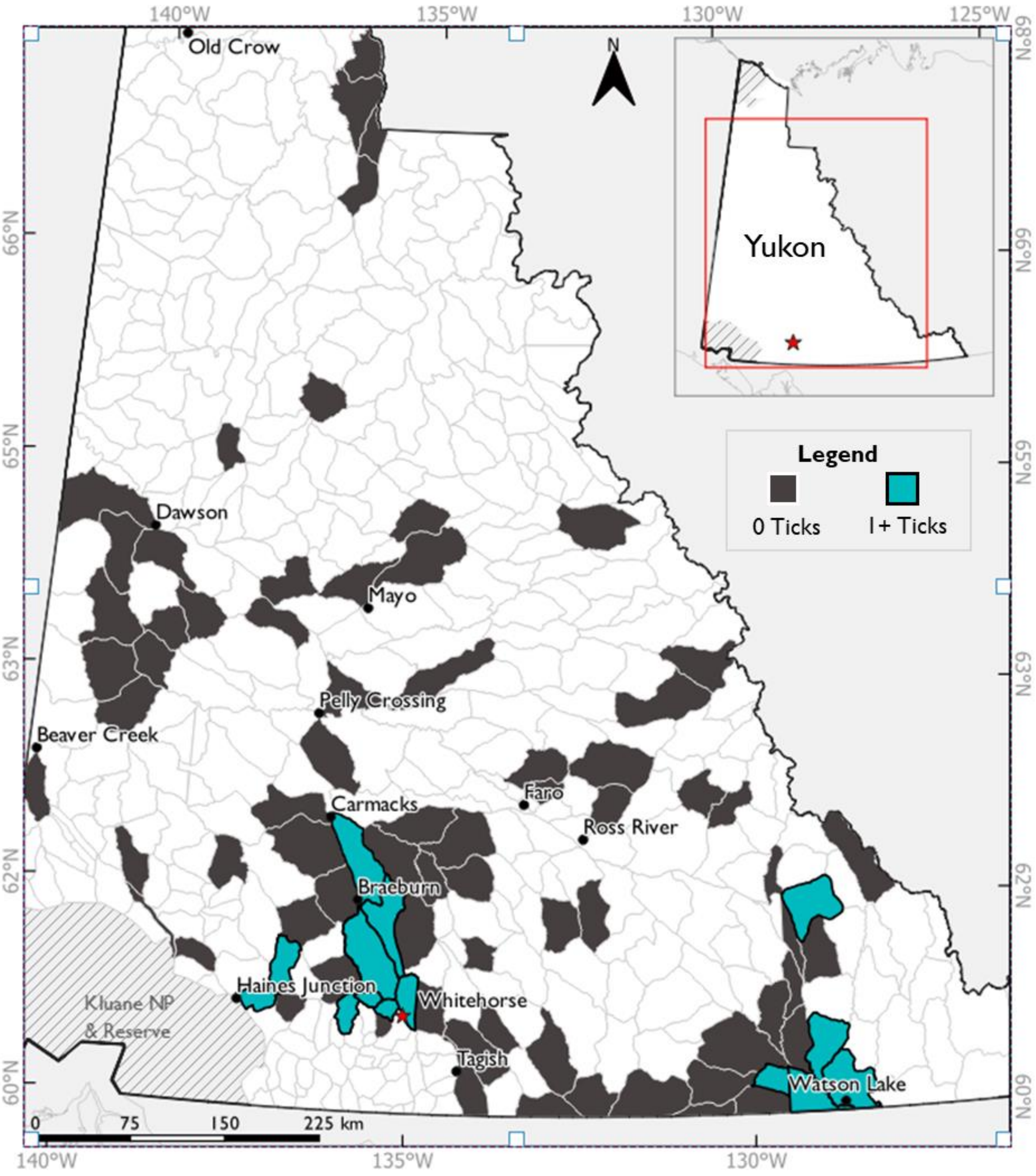

Figure S3. Map of Yukon Game Management Subzones (GMS) where hide samples have been received from cervid hunters (moose, elk, caribou, mule deer), 2011 – 2020 seasons. Subzones where one or more winter ticks (*Dermacentor albipictus)* have been found on one or more hides are shown in blue, all hides on which ticks have not yet been detected are given in dark grey. GMS that have not been sampled are shown in white.
